# Maximizing Knee OA Treatment: A Comparative Look at Physiotherapy and Injections

**DOI:** 10.3390/jpm14111077

**Published:** 2024-10-26

**Authors:** Danilo Donati, Federica Giorgi, Tarantino Domiziano, Luigi Tarallo, Fabio Catani, Daniela Platano, Roberto Tedeschi

**Affiliations:** 1 Clinical and Experimental Medicine PhD Program, University of Modena and Reggio Emilia, 41125 Modena, Italy; 2 Physical Therapy and Rehabilitation Unit, Policlinico di Modena, 41125 Modena, Italy; 3 Pediatric Physical Medicine and Rehabilitation Unit, IRCCS Institute of Neurological Sciences, 40126 Bologna, Italy; 4 IRCCS Fondazione Don Carlo Gnocchi ONLUS, 50143 Florence, Italy; 5 Department of Public Health, University of Naples Federico II, 80131 Naples, Italy; 6 Department of Orthopedics and Traumatology, Polyclinic of Modena, University of Modena and Reggio Emilia, 41125 Modena, Italy; 7 Department of Biomedical and Neuromotor Sciences, Alma Mater Studiorum, University of Bologna, 40127 Bologna, Italy; 8 Physical Medicine and Rehabilitation Unit, IRCCS Istituto Ortopedico Rizzoli, 40136 Bologna, Italy

**Keywords:** knee osteoarthritis, physiotherapy, intra-articular injections, pain management, functional recovery

## Abstract

Background: Knee osteoarthritis (OA) is a prevalent and disabling condition often managed with physiotherapy or intra-articular injections. However, the comparative effectiveness of these treatments remains unclear. This systematic review aimed to evaluate and compare the efficacy of physiotherapy and intra-articular injections in managing knee OA. Methods: A systematic search of PubMed, Scopus, Web of Science, PEDro, and Cochrane Library was conducted. Randomized controlled trials (RCTs) comparing physiotherapy and intra-articular injections in knee OA patients were included. Key outcomes included pain (VAS), function (WOMAC, KOOS), range of motion (ROM), and quality of life. Data from five studies with a total of 552 participants were analyzed. Results: Intra-articular injections, particularly botulinum toxin and hyaluronic acid, were found to provide rapid pain relief, outperforming physiotherapy in short-term pain management. However, physiotherapy contributed significantly to long-term functional improvements, particularly in early-stage OA. Combination therapy of injections and physiotherapy yielded the best short-term pain relief and functional outcomes. Heterogeneity in study designs and follow-up periods limited the generalizability of findings. Conclusions: Intra-articular injections are effective for immediate pain control, while physiotherapy plays a crucial role in maintaining joint function, especially for long-term management. Combining both interventions may offer the most comprehensive benefits. Further research is needed to determine the long-term efficacy of these treatments.

## 1. Introduction

Osteoarthritis (OA) is a chronic, degenerative joint disease that primarily affects the synovial joints. The most commonly impacted sites are the knee, hip, spine, and small joints of the hands and feet. OA is characterized by focal areas of articular cartilage loss within the joint, accompanied by hypertrophy of subchondral bone (osteophytes and subchondral sclerosis) and thickening of the joint capsule [1,2,3,4]. Additionally, nearby soft tissues, such as the synovium, may exhibit inflammatory infiltrates, ligaments may become lax, and the surrounding musculature may weaken, further contributing to joint dysfunction [2,5,6]. The American College of Rheumatology (ACR) classifies OA into two categories, idiopathic (primary) OA, which affects patients without any pre-existing diseases, and secondary OA, which occurs in association with a pre-existing condition. Idiopathic OA can be further subdivided into localized, affecting a single joint, and generalized, involving three or more joints simultaneously. OA is more prevalent in countries with higher socioeconomic status and longer life expectancy [4,7]. However, epidemiological data often rely on radiographic surveys that are less sensitive in detecting early-stage disease, leading to an underestimation of the actual prevalence [4]. The primary symptom of OA is pain [8], which typically occurs during movement or after physical activity and improves with rest. Morning stiffness is also common, lasting for no more than 30 min [2,9,10]. As the disease progresses, functional limitations develop, which can significantly impair the activities of daily living. 

In advanced cases, joint deformities become visible, particularly in the interphalangeal joints of the hands [11,12,13]. Additionally, some patients report an increase in pain associated with changes in weather conditions or humidity [14,15,16,17]. Several risk factors contribute to the development of OA, both genetic and mechanical. Age is a critical risk factor, although the mechanisms underlying its impact remain unclear. Biological changes, tissue adaptations to biomechanical stress, and reduced joint resilience due to sarcopenia are thought to play a role [12,18,19,20,21]. Gender differences have been noted, with women, especially post-menopausal women, being at a higher risk of developing knee OA due to hormonal changes that affect joint health. Other key risk factors include female gender, particularly after menopause, and genetic predisposition, which may account for up to 40% of the risk of developing knee OA [22,23,24,25,26]. Mechanical risk factors include obesity [9], which has been strongly linked to an increased risk of OA development, and joint malalignment, which disrupts normal load distribution across the joint [27,28]. Additionally, joint injuries and certain occupations that involve repetitive strain on the knees, such as prolonged squatting or heavy lifting, further exacerbate the risk. Knee OA, or gonarthrosis, is the most common form of OA in the elderly population, with an estimated 250 million people affected globally. There is no significant evidence to suggest that OA affects the right knee more than the left knee; however, individual cases may vary depending on factors such as joint use and injury history. The knee is particularly susceptible due to its role in weight-bearing and frequent use in daily activities, making it more prone to wear and tear over time.

The disease is characterized by progressive cartilage loss, leading to pain, reduced range of motion (ROM), and muscle weakness. Diagnosis is typically made through radiographic evaluation, although MRI and CT scans are used to assess soft tissue involvement. In advanced stages, joint deformities such as varus or valgus alignment can occur, further impairing mobility [29]. Diagnosis is typically made through radiographic evaluation, although MRI and CT scans are used to assess soft tissue involvement. However, the disease is often detected only in its advanced stages, as early signs are difficult to identify radiographically [30,31]. There is no definitive cure for OA, and current treatment strategies focus on alleviating symptoms and slowing disease progression. Treatment options are typically categorized as either conservative or surgical. Conservative approaches include physiotherapy [32,33,34,35,36,37,38,39], lifestyle modifications, pharmacotherapy, and intra-articular injections [40,41,42,43] of substances such as corticosteroids, hyaluronic acid, or platelet-rich plasma (PRP). Surgical interventions, including total or partial knee replacements, are reserved for patients in advanced stages of the disease when conservative measures fail to provide adequate relief [44,45]. While physiotherapy and intra-articular injections are both commonly used in managing knee OA, there remains considerable debate regarding their relative efficacy. Several studies have explored the impact of these interventions on pain and functional outcomes, yet there is no consensus on which approach offers superior benefits. The lack of uniformity in study designs, patient populations, and treatment protocols adds to the complexity of drawing definitive conclusions from the existing literature. The objective of this systematic review is to critically assess the effectiveness of physiotherapy compared to intra-articular injections in the management of knee osteoarthritis. By synthesizing the available evidence, this review aims to provide clearer guidance on the most effective treatment strategies for improving pain, function, and quality of life in patients with knee OA, thereby contributing to more informed clinical decision making.

## 2. Methods

The present scoping review was conducted following the JBI methodology [46,47] for scoping reviews. The Preferred Reporting Items for Systematic reviews and Meta-Analyses extension for Scoping Reviews (PRISMA-ScR) [48] checklist for reporting was used. 

Review question

We formulated the following research question: “Is physiotherapy more effective than intra-articular injections in reducing pain and improving function and quality of life in patients with knee osteoarthritis?”

Eligibility criteria

Studies were eligible for inclusion if they met the following Population, Concept, and Context (PCC) criteria.

Population (P): Adults diagnosed with knee osteoarthritis (gonarthrosis) based on clinical or radiographic criteria. This includes both male and female patients of varying ages, typically over 50, who experience symptomatic osteoarthritis in the knee joint.

The severity of knee osteoarthritis could range from mild to severe, as categorized by the Kellgren and Lawrence grading scale or other accepted diagnostic criteria.

We included patients with either idiopathic (primary) or secondary osteoarthritis, excluding those with other forms of arthritis (e.g., rheumatoid arthritis).

Concept (C): This review focuses on comparing the effectiveness of physiotherapy versus intra-articular injections in managing knee osteoarthritis.

Physiotherapy includes various rehabilitation interventions such as exercise programs (strengthening, stretching, range of motion), manual therapy, electrotherapy, and patient education aimed at improving joint function and reducing pain.

Intra-articular injections include treatments such as hyaluronic acid (HA), corticosteroids, platelet-rich plasma (PRP), and other injectables designed to alleviate pain, reduce inflammation, or improve joint lubrication.

The primary outcomes of interest include the following:

- Pain reduction (as measured by the Visual Analog Scale (VAS) or other validated pain scales).
- Improvement in function (as assessed by standardized measures such as the WOMAC (Western Ontario and McMaster Universities Arthritis Index) or similar functional outcome measures).
- Quality of life improvements related to knee function and mobility.

Context (C): Studies that evaluated treatments in clinical, outpatient, or rehabilitation settings wherein patients received either physiotherapy or intra-articular injections for knee osteoarthritis.

This review considers studies conducted in a variety of geographical locations and healthcare systems, without restriction to specific countries or regions.

Randomized controlled trials (RCTs), quasi-experimental studies, and cohort studies were included, provided they directly compared physiotherapy to intra-articular injections.

The timeframes for follow-up could vary, but studies needed to have reported outcomes within a defined follow-up period (e.g., 1, 3, or 6 months or longer).

Exclusion criteria

Studies that did not meet the specific PCC criteria were excluded.

Search strategy

An initial limited search of MEDLINE was performed through the PubMed interface to identify articles on the topic, and then the index terms used to describe the articles were used to develop a comprehensive search strategy for MEDLINE. The search strategy, which included all identified keywords and index terms, was adapted for use in Cochrane Central, Scopus, PEDro, and Web of Science. In addition, gray literature and reference lists of all relevant studies were also searched. Searches were conducted on 31 July 2024 with no date limitation.

PubMed: ((“Osteoarthritis, Knee”[Mesh] OR “knee osteoarthritis”) AND (“Physiotherapy”[Mesh] OR “Physical Therapy Modalities”[Mesh] OR “Rehabilitation” OR “exercise therapy”) AND (“Injections, Intra-Articular”[Mesh] OR “Hyaluronic Acid”[Mesh] OR “corticosteroid injection” OR “Platelet-Rich Plasma” OR “PRP”) AND (“Pain”[Mesh] OR “pain management” OR “Quality of Life”[Mesh] OR “functional improvement” OR “function”))

Scopus: (TITLE-ABS-KEY(“knee osteoarthritis” OR “gonarthrosis”) AND TITLE-ABS-KEY(“physiotherapy” OR “physical therapy” OR “rehabilitation” OR “exercise therapy”) AND TITLE-ABS-KEY(“intra-articular injections” OR “hyaluronic acid” OR “corticosteroid injection” OR “Platelet-Rich Plasma” OR “PRP”) AND TITLE-ABS-KEY(“pain management” OR “quality of life” OR “functional improvement”))

Cochrane: ((“knee osteoarthritis” OR “gonarthrosis”) AND (“physiotherapy” OR “physical therapy” OR “rehabilitation” OR “exercise therapy”) AND (“intra-articular injections” OR “hyaluronic acid” OR “corticosteroid injection” OR “Platelet-Rich Plasma” OR “PRP”) AND (“pain” OR “quality of life” OR “function”))

Web of Science: TS = (“knee osteoarthritis” OR “gonarthrosis”) AND TS = (“physiotherapy” OR “physical therapy” OR “rehabilitation” OR “exercise therapy”) AND TS = (“intra-articular injections” OR “hyaluronic acid” OR “corticosteroid injection” OR “Platelet-Rich Plasma” OR “PRP”) AND TS = (“pain management” OR “quality of life” OR “functional improvement”)

Pedro: (“knee osteoarthritis” OR “gonarthrosis”) AND (“physiotherapy” OR “physical therapy” OR “rehabilitation” OR “exercise therapy”) AND (“intra-articular injections” OR “hyaluronic acid” OR “corticosteroid injection” OR “Platelet-Rich Plasma” OR “PRP”)

Study selection

The process described involved a systematic approach to selecting studies for a scoping review. Initially, search results were collected and refined using Zotero, with duplicates removed. The screening involved two levels: title and abstract review, followed by full-text assessment, both conducted independently by two authors and discrepancies resolved by a third. The selection adhered to the PRISMA 2020 guidelines, ensuring transparency and reliability. This rigorous methodology aimed to identify relevant articles that directly address the research question, maintaining a comprehensive and systematic approach in the review process.

Data extraction and data synthesis

Data extraction for the scoping review was carried out using a form based on the JBI tool, capturing crucial details like authorship, publication country and year, study design, patient characteristics, outcomes, interventions, procedures, and other relevant data. Descriptive analyses of these data were conducted, with results presented numerically to show study distribution. The review process was clearly mapped for transparency, and data were summarized in tables for easy comparison and understanding of the studies’ key aspects and findings.

## 3. Results

**Figure 1:**
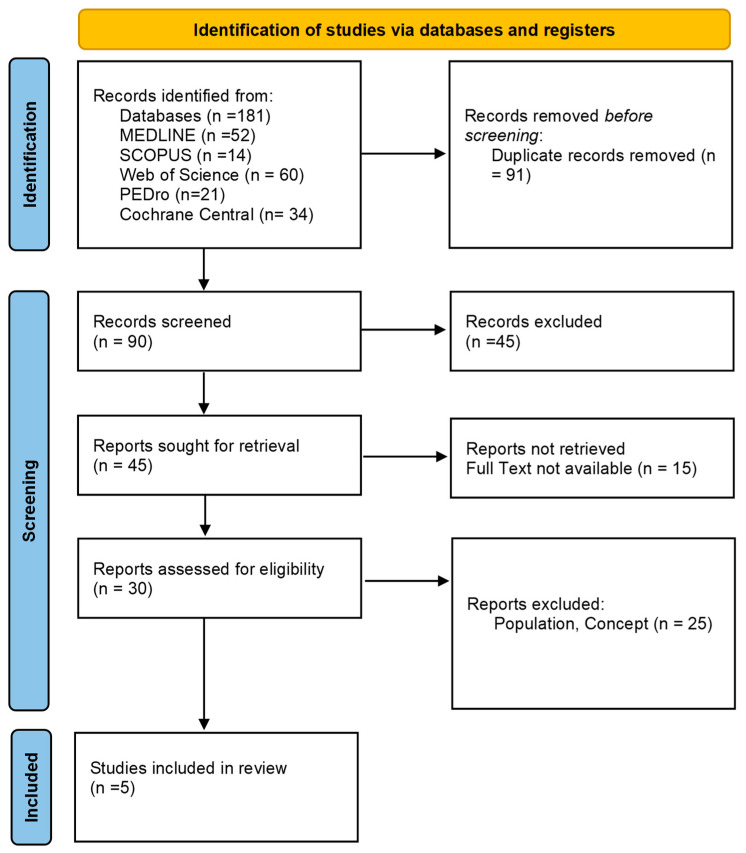
Preferred Reporting Items for Systematic reviews and Meta-Analyses 2020 (PRISMA) flow diagram.

As presented in the PRISMA 2020 flow diagram (Figure 1), from 181 records identified by the initial literature searches, 176 were excluded and 5 articles were included (Table 1 and Table 2). The quality of the studies was assessed with the PEDro scale and ROB2 (Table 3).

### 3.1. Pain Reduction (VAS—Visual Analog Scale)

- Rezasoltani Z, 2020 [49]:
  ○ All groups (physical therapy, botulinum toxin type A, hyaluronic acid, and dextrose) showed significant reductions in pain as measured by VAS. The botulinum toxin type A and dextrose groups exhibited the greatest reductions in pain compared to the physical therapy and hyaluronic acid groups. Specifically, Group 3 (hyaluronic acid) had significantly lower VAS scores than Groups 1 (physical therapy), 2 (botulinum toxin type A), and 4 (dextrose) (*p* < 0.05). Additionally, Group 2 (botulinum toxin) showed better results than Group 1 (physical therapy) (*p* = 0.015).
- Saccomanno MF, 2016 [50]:
  ○ Significant reductions in pain were observed in all three groups (hyaluronic acid, exercise-based rehabilitation (EBR), and combined treatment). The group that received both hyaluronic acid and EBR (Group 3) showed the largest reduction in pain compared to the hyaluronic acid-only group (*p* = 0.043 at T1). Pain in the EBR-only group deteriorated at T3 compared to T1 (*p* < 0.004).
- Bao X, 2018 [51]:
  ○ Both the botulinum toxin type A group and the hyaluronate group demonstrated significant reductions in pain according to VAS compared to the saline injection control group. Group 2 (botulinum toxin type A) was significantly more effective in reducing pain compared to Groups 1 and 3 (hyaluronate) at both T1 (*p* < 0.05) and T2 (*p* < 0.05).
- Karatosun V, 2009 [52]:
  ○ Both groups (hyaluronic acid and exercise program) showed significant pain reduction, particularly at rest and during transfers. The exercise group (G2) was significantly better at reducing pain during transfers compared to the hyaluronic acid group (T1-T4). However, for pain during activities, the hyaluronic acid group (G1) performed better at T4 (*p* = 0.039) and T5 (*p* = 0.001).
- Kawasaki T, 2009 [53]:
  ○ Both the exercise and hyaluronic acid groups demonstrated significant reductions in pain (VAS). No significant difference was found between the two groups at the 6-month follow-up (T6) (*p* = 0.171).

### 3.2. Function (WOMAC and KOOS Scores)

- Rezasoltani Z, 2020 [49]:
  ○ The KOOS scores (including subdomains of symptoms, stiffness, pain, function in daily activities, sports, and quality of life) showed improvement in all groups. Group 3 (hyaluronic acid) had significantly lower KOOS scores compared to Groups 1 (physical therapy), 2 (botulinum toxin type A), and 4 (dextrose) (*p* < 0.05). Group 2 (botulinum toxin type A) demonstrated better outcomes in KOOS subgroups—particularly in reducing symptoms, pain, sports function, and quality of life—than the physical therapy group (*p* < 0.001).
- Saccomanno MF, 2016 [50]:
  ○ All groups demonstrated improvements in WOMAC scores (pain, stiffness, and function). Group 3 (hyaluronic acid + EBR) had better pain outcomes than Group 1 (hyaluronic acid alone) (*p* < 0.043 at T1). Stiffness in the EBR group (G2) worsened from T1 to T3 (*p* = 0.026), and the EBR group also saw a deterioration in function between T1 and T3 (*p* = 0.025). No significant differences were found in active range of motion (AROM) between the groups.
- Bao X, 2018 [51]:
  ○ Both Group 2 (botulinum toxin type A) and Group 3 (hyaluronate) had significantly improved WOMAC scores in pain and function compared to Group 1 (saline). Group 2 showed significantly better results in WOMAC pain and function than Group 3 at T1 (*p* < 0.05) and T2 (*p* < 0.05). Group 2 also showed significantly better SF-36 scores for quality of life at both T1 and T2 than the other groups (*p* < 0.01).
- Karatosun V, 2009 [52]:
  ○ Significant improvements in function were observed in both groups, as measured by the HSS knee score. The exercise group (G2) showed better results in transfer activities than the hyaluronic acid group at T1-T4, but at T5, the hyaluronic acid group demonstrated better performance in daily activities (*p* < 0.039) and walking distance (*p* = 0.012).
- Kawasaki T, 2009 [53]:
  ○ Both groups (exercise and hyaluronic acid) showed improvements in function as measured by JKOM (Japanese Knee Osteoarthritis Measure) and OMERACT-OARSI scores. However, no significant difference was observed between the two groups at T6 (*p* = 0.721 for JKOM, *p* = 0.767 for OMERACT-OARSI).

### 3.3. Range of Motion (ROM)

- Rezasoltani Z, 2020 [49]:
  ○ No specific ROM measurements were reported, but improvements in function were noted in the KOOS subdomains, including ROM-related activities like sports.
- Saccomanno MF, 2016 [50]:
  ○ No significant changes in active ROM were reported in any of the groups. AROM remained unchanged across the hyaluronic acid, EBR, and combined treatment groups (*p* > 0.05).
- Bao X, 2018 [51]:
  ○ No specific results for ROM were reported, but improvements in function and daily activities imply some level of ROM enhancement.
- Karatosun V, 2009 [52]:
  ○ Both groups demonstrated significant improvements in ROM, particularly in knee flexion. However, the hyaluronic acid group showed better ROM improvements at T5 (*p* = 0.000), and the overall HSS score, which includes ROM, was higher in this group at T5 (*p* = 0.023).
- Kawasaki T, 2009 [53]:
  ○ Both groups (exercise and hyaluronic acid) showed improvements in ROM, particularly in early stage OA. ROM improvement was not statistically significant between the groups in advanced OA (*p* = 0.094), but for early OA, ROM improvements were comparable (*p* = 0.879).

### 3.4. Swelling and Joint Space Width (JSW)

- Kawasaki T, 2009 [53]:
  ○ The exercise group showed significantly greater reductions in joint swelling compared to the hyaluronic acid group in early stage OA (*p* = 0.019). In advanced OA, no significant differences were observed between the two groups (*p* = 0.571). Joint space width (JSW) was not significantly different between the groups, indicating no major structural changes during the 6-month follow-up.

### 3.5. Quality of Life (SF-36 and KOOS QOF)

- Rezasoltani Z, 2020 [49]:
  ○ Group 2 (botulinum toxin type A) showed the best improvement in the quality of life (QOF) subdomain of the KOOS score compared to the other groups (*p* < 0.001). Group 2 also outperformed Group 4 (dextrose) in reducing symptoms and improving quality of life (*p* < 0.004).
- Saccomanno MF, 2016 [50]:
  ○ Quality of life was measured through WOMAC, but specific QOF results were not separately reported.
- Bao X, 2018 [51]:
  ○ Group 2 (botulinum toxin type A) showed significant improvements in SF-36 quality of life scores compared to Group 1 (saline) and Group 3 (hyaluronate) at T1 (*p* < 0.01) and T2 (*p* < 0.01). Group 2 had the highest SF-36 scores across all follow-up points.

### 3.6. Biochemical Markers

- Kawasaki T, 2009 [53]:
  ○ There were no significant differences between the exercise and hyaluronic acid groups in terms of biochemical markers. Both groups showed similar results at T6 for markers of inflammation and cartilage degradation.

## 4. Discussion

The aim of this review was to evaluate the effectiveness of physiotherapy compared to intra-articular injections in the treatment of knee osteoarthritis (OA). However, the available literature offers limited direct comparisons between these two therapeutic approaches. Upon reviewing the selected studies, several issues became apparent, particularly in relation to the variability in participant characteristics, interventions, outcome measures, and follow-up durations. This heterogeneity complicates the ability to generalize findings or draw robust conclusions. The total participant count across all included studies amounted to 552, with a clear predominance of female subjects (422 women to 130 men). Most studies provided detailed descriptions of the participants’ ages and treatment groups, and nearly all used the Kellgren and Lawrence classification to specify the severity of knee OA among participants, except for one study. This inconsistency further contributes to the difficulty in comparing results across different interventions and study designs.

A key aspect of the studies was the inclusion of exercise programs, which featured prominently in most of the interventions. In two studies, all participants engaged in exercise regimens aimed at improving knee function. The exercise protocols varied in intensity and frequency, with one study prescribing daily isometric quadriceps and gastrocnemius/soleus stretching, while the other study incorporated strengthening and balance exercises over five weekly sessions for eight weeks. The remaining studies assigned exercise programs only to specific treatment groups, with protocols ranging from isolated exercise sessions to more comprehensive programs integrated with other treatments, such as hyaluronic acid injections [40,54]. These variations in exercise intervention approaches highlight the challenge of standardizing treatment comparisons across studies. When examining the efficacy of different treatments, the study by Rezasoltani et al. [49] stands out for its comparison of physical therapy with three different types of intra-articular injections—botulinum toxin, hyaluronic acid, and dextrose. While all treatments demonstrated effectiveness in managing knee OA, the injection groups, particularly those receiving botulinum toxin and dextrose, showed more substantial and sustained pain relief over a three-month period. This finding suggests that intra-articular injections, especially botulinum toxin, may provide more immediate and longer-lasting benefits in pain control than physical therapy [55,56,57]. However, the broader implications for long-term function recovery remain unclear, as physical therapy was not as effective in reducing pain but might still play a role in overall functional improvement. The term ‘long-term’ in this study refers to follow-up periods extending to 6 months and beyond, as indicated by the included studies, with one study following patients for up to 18 months. Similarly, in the work of Saccomanno et al. [50], the efficacy of hyaluronic acid injections and knee-specific exercise programs was compared, both separately and in combination. This study further reinforces the idea that a multi-modal approach may offer more comprehensive benefits. While all treatment groups exhibited early improvements in pain and function, the combination of hyaluronic acid injections and exercise yielded the most pronounced short-term pain relief. However, this effect diminished over time, and by the six-month follow-up, the benefits had substantially waned. The exercise-only group, in contrast, showed a notable deterioration in pain and function by the study’s end, underscoring the potential limitations of exercise when used in isolation. Despite these findings, Saccomanno et al. concluded that the integrated treatment approach was most effective in achieving short-term pain reduction, although longer-term effectiveness remained uncertain. Further supporting these findings, Bao et al. [51] focused on the combination of therapeutic exercise following injections of either botulinum toxin type A or hyaluronate [58,59,60]. The results were particularly compelling for the botulinum toxin group, which demonstrated significant improvements in both pain and functional outcomes. Bao’s study suggests that reducing pain through injections may facilitate better patient adherence to exercise programs, as participants experiencing less pain are more likely to engage in and benefit from rehabilitation. Interestingly, the botulinum toxin group consistently outperformed the hyaluronate group in terms of pain relief and functional gains, suggesting that this particular injection may offer superior benefits for short-term symptom management.

In contrast, Karatosun et al. [52] explored the long-term outcomes of hyaluronic acid injections versus a progressive six-week exercise program. Although both groups exhibited significant improvements in pain and function, the study’s long-term follow-up (18 months) revealed minimal differences between the two approaches. This suggests that while short-term variations in treatment efficacy may exist, the long-term benefits of hyaluronic acid injections and exercise programs may converge. Therefore, Karatosun’s findings support the use of either treatment modality, particularly for patients seeking symptom relief and functional improvement over an extended period. Finally, Kawasaki et al. [53] focused on a cohort of post-menopausal women with knee OA, comparing the effects of hyaluronic acid injections and an exercise program. Both treatments significantly reduced pain and improved function, with no major differences between the two groups at the six-month follow-up. However, Kawasaki’s study introduced an important distinction between early-stage and advanced OA, demonstrating that exercise was particularly effective in reducing swelling and improving ROM in patients with early-stage OA, while the benefits in advanced OA were less pronounced. Hyaluronic acid injections primarily provide lubrication and cushioning within the joint, whereas botulinum toxin injections act by reducing muscle spasms and pain. Both can be effective, but botulinum toxin may offer more immediate pain relief in some cases. This nuanced finding underscores the importance of tailoring treatment strategies to disease severity, with early interventions, such as exercise, potentially offering greater benefits for long-term joint health and mobility. While previous studies have explored the effectiveness of physiotherapy and injection therapy for managing knee osteoarthritis, this study provides a novel approach by directly comparing the short-term and long-term outcomes of these treatments across multiple dimensions, such as pain relief, functional recovery, and quality of life, in a broader population. Additionally, the integration of combination therapy (physiotherapy and injections) in this analysis offers unique insights into its potential for synergistic benefits, which has been less explored in prior research. The heterogeneity of follow-up durations and the variability in patient characteristics across existing studies make direct comparisons challenging, but this study attempts to standardize these variables, providing a more robust evaluation of treatment outcomes.

This review highlights several limitations. First, the heterogeneity in the study designs, populations, and interventions across the included studies makes direct comparisons challenging. The variation in OA severity, exercise protocols, and injection types introduces confounding factors. Additionally, the studies used different primary and secondary outcome measures, and follow-up periods varied widely, ranging from 8 weeks to 18 months, which limits the ability to draw definitive conclusions on long-term efficacy. The predominance of female participants may also limit the generalizability of the results to a broader population. Finally, the relatively small sample sizes in some studies and the lack of blinding in certain trials raise concerns about potential biases in the results. 

### Implications for Clinical Practice 

The findings of this review suggest several important clinical implications for the management of knee osteoarthritis (OA). Both physiotherapy and intra-articular injections offer valuable treatment options, but their use should be tailored to the patient’s specific condition and treatment goals.

1. Intra-articular injections, particularly botulinum toxin and hyaluronic acid, can provide rapid pain relief, making them suitable for patients with moderate to severe pain. These injections may also facilitate better patient compliance with subsequent rehabilitation programs by reducing discomfort during exercise. Intra-articular injections carry some risks, including infection, bleeding, allergic reactions, and, in rare cases, damage to the joint. Repeated injections may also contribute to cartilage degradation over time.
2. Physiotherapy, while not as effective for immediate pain control, plays a crucial role in long-term functional recovery, especially in patients with early-stage OA. Regular exercise programs that focus on strengthening, range of motion, and proprioception can help maintain joint mobility and reduce the risk of progression.
3. Combination therapy, integrating both intra-articular injections and physiotherapy, appears to offer optimal short-term benefits, particularly in pain reduction and improving quality of life. This approach may be ideal for patients who need immediate relief but also aim for long-term functional improvements.
4. Early intervention, particularly with exercise-based rehabilitation, is critical in managing early-stage OA. Patients with mild symptoms may benefit more from non-invasive physiotherapy interventions, delaying or avoiding the need for injections or surgical interventions.

## 5. Conclusions

This review highlights the complementary roles of physiotherapy and intra-articular injections in managing knee osteoarthritis. Injections, particularly botulinum toxin and hyaluronic acid, offer effective short-term pain relief, while physiotherapy supports long-term functional recovery, especially in early stage OA. Combining both treatments may provide optimal results, but individual treatment should be tailored to the patient’s needs and disease severity. Further research is needed to explore the long-term efficacy of and best practices for integrating these therapies in clinical care.

## Figures and Tables

**Table 1:** Main characteristics of included studies and summary of the studies included in the systematic review comparing the effectiveness of physiotherapy and intra-articular injections in patients with knee osteoarthritis, detailing study design, country, sample size, participant characteristics, interventions, and outcomes with follow-up periods.

Author and Year	Study Design	Country	Sample Size and Participant Characteristics	Intervention and Control	Outcomes and Follow-Up
Rezasoltani Z, 2020 [49]	RCT	Tehran, Iran	N (M/F) = 120 (45/75); Age ≥ 50 years; Group 1 (*n* = 30), mean age 70 (6.3); Group 2 (*n* = 30), mean age 67.7 (7.3); Group 3 (*n* = 30), mean age 66.1 (9.1); Group 4 (*n* = 30), mean age 64.8 (5.8); diagnosis of knee osteoarthritis according to ACR criteria (Grade 3–4 on the Kellgren and Lawrence scale).	All participants followed a daily 30 min exercise program. Group 1 (G1): physical therapies (20 min of superficial heat, transcutaneous nerve stimulation, 5 min of ultrasound); Group 2 (G2): single injection of botulinum toxin type A; Group 3 (G3): three hyaluronic acid injections (one per week); Group 4 (G4): three dextrose injections (one per month).	VAS (pain), KOOS (symptoms, stiffness, pain, function in daily activities and sports, quality of life QOF). Follow-up: 3 months (T1: 1 week, T2: 4 weeks, T3: 3 months).
Saccomanno MF, 2016 [50]	RCT	Rome, Italy	N (M/F) = 165 (44/121); Age ≥ 18 years; Group 1 (*n* = 55), mean age 62.8 (13.2); Group 2 (*n* = 55), mean age 61.2 (10.1); Group 3 (*n* = 55), mean age 61.4 (9.7); diagnosis of knee osteoarthritis (excluding Grade IV on the Kellgren and Lawrence scale and Iwano system); good general health conditions.	Group 1 (G1): received three hyaluronic acid injections (one every two weeks); Group 2 (G2): exercise-based rehabilitation program (EBR) for the knee (20 sessions in one month); Group 3 (G3): received both treatments (hyaluronic acid injections and EBR).	WOMAC (pain, stiffness, function), ROM (flexion and extension). Follow-up: 6 months (T1: 1 month, T2: 3 months, T3: 6 months).
Bao X, 2018 [51]	RCT	Shao-Guan, China	N (M/F) = 60 (26/34); Age range: 59–72 years; Group 1 (*n* = 20), mean age 65.3 (3.52); Group 2 (*n* = 20), mean age 66.4 (3.49); Group 3 (*n* = 20), mean age 66.0 (2.09); diagnosis of knee osteoarthritis (Grade II or higher on the Kellgren and Lawrence scale); VAS pain score ≥6 after walking continuously for 100 m on flat terrain.	All participants followed the same exercise protocol (5 sessions per week for 8 weeks); Group 1 (G1, control): saline injection; Group 2 (G2): single injection of botulinum toxin type A; Group 3 (G3): hyaluronate injection (once per week for 5 weeks).	WOMAC, VAS (pain), SF-36. Follow-up: 2 months (T1: 4 weeks, T2: 8 weeks).
Karatosun V, 2009 [52]	Prospective RCT	Izmir, Turkey	N (M/F) = 105 (15/90); Mean age 56 years; Group 1 (*n* = 52), mean age 57.8 (12.1); Group 2 (*n* = 53), mean age 55.3 (13.6); Diagnosis of knee osteoarthritis according to ACR criteria (Grade III on the Kellgren and Lawrence scale).	Group 1 (G1): received three hyaluronic acid injections (one per week); Group 2 (G2): progressive knee exercise program for 6 weeks.	HSS knee score (pain, function, ROM, strength, flexion deformity, instability, contractures); Pain at rest, pain while climbing stairs, pain during transfers, walking distance, ROM. Follow-up: 18 months (T1: 1 week, T2: 2 weeks, T3: 3 weeks, T4: 6 weeks, T5: 3 months, T6: 6 months, T7: 12 months, T8: 18 months).
Kawasaki T, 2009 [53]	Prospective RCT	Japan	N (M/F) = 102 (0/102); Age > 50 years (post-menopausal women); Group 1 (*n* = 52), mean age 71.2 (7.1); Group 2 (*n* = 50), mean age 69.5 (8.4); diagnosis of medial femorotibial compartment knee osteoarthritis according to ACR criteria.	Group 1 (G1): knee exercise program (isometric exercises and ROM exercises); Group 2 (G2): hyaluronic acid injection (once per week for 5 weeks).	VAS, JKOM, OMERACT-OARSI (pain/function), ROM, swelling, joint space width (JSW), biochemical markers. Follow-up: 6 months (T1: 4 weeks, T2: 8 weeks, T3: 12 weeks, T4: 16 weeks, T5: 20 weeks, T6: 24 weeks).

Legend: ACR: American College of Rheumatology, ADL: activities of daily living, EBR: exercise-based rehabilitation, G1: Group 1, G2: Group 2, G3: Group 3, G4: Group 4, HSS: Hospital for Special Surgery Knee Score, JKOM: Japanese Knee Osteoarthritis Measure, KOOS: Knee Injury and Osteoarthritis Outcome Score, PRP: platelet-rich plasma, QOF: quality of life, ROM: range of motion, SF-36: Short-Form Health Survey, VAS: visual analog scale, WOMAC: Western Ontario and McMaster Universities Osteoarthritis Index, JSW: joint space width, OMERACT: Outcome Measures in Rheumatology.

**Table 2:** Table of study comparisons showing a comparison of outcomes in different treatment groups from the studies included in the systematic review on knee osteoarthritis. Each group was treated with different interventions such as physical therapy, botulinum toxin type A injections, hyaluronic acid, and dextrose. Results are summarized in terms of pain relief (VAS), function (WOMAC, KOOS), range of motion (ROM), and other key outcome measures across various follow-up periods.

Author	Group 1 (G1)	Group 2 (G2)	Group 3 (G3)	Group 4 (G4)	Comparison
Rezasoltani Z, 2020 [49]	Physical therapy VAS + (*p* < 0.001)	Botulinum toxin type A VAS + (*p* < 0.001)	Hyaluronic acid VAS +	Dextrose VAS + (*p* < 0.001)	VAS: G3 < G1, G2, G4 (*p* < 0.05); G2 > G1 (*p* = 0.015). KOOS: G3 < G1, G2, G4 (*p* < 0.05); G2 = G1 (*p* = 0.254); G4 = G1 (*p* = 0.097). KOOS subgroups: G2 > G1 (*p* < 0.001 for symptoms, pain, sport, quality of life (QOF)); G2 > G4 (*p* < 0.004 for symptoms).
Saccomanno MF, 2016 [50]	Hyaluronic acid WOMAC pain + (*p* < 0.0001)	Exercise-based rehabilitation (EBR) WOMAC pain + (*p* < 0.0001)	EBR + Hyaluronic acid WOMAC pain + (*p* < 0.0001)	—	WOMAC pain: G3 > G1 (T1, *p* = 0.043); WOMAC stiffness: G2 at T3 < G2 at T1 (*p* < 0.004); G3 at T3 < G3 at T1 (*p* = 0.001); WOMAC function: G1 = G2 = G3; G2 at T3 < G2 at T1 (*p* = 0.025); AROM: G1 = G2 = G3.
Bao X, 2018 [51]	Saline injection WOMAC − VAS − SF-36	Botulinum toxin type A WOMAC + VAS + SF-36	Hyaluronate WOMAC + VAS + SF-36	—	WOMAC/VAS: G2 > G1, G2 > G3 (T1, *p* < 0.05); G1 = G3 (T1, *p* > 0.05). WOMAC/VAS: G2 > G1, G3 (T2, *p* < 0.05). SF-36: G2 > G1 (T1, *p* < 0.01); G2 > G3 (T1, *p* < 0.05); G1 = G3 (T1, *p* = 0.29); G2 > G1, G3 (T2, *p* < 0.01).
Karatosun V, 2009 [52]	Hyaluronic acid HSS +; Pain at rest +; Pain while climbing stairs +; Pain during transfers +	Exercise program HSS +; Pain during transfers + (T1, *p* = 0.042; T2, *p* = 0.000; T3, *p* = 0.010; T4, *p* = 0.024)	—	—	Pain during transfers: G2 > G1 (T1-T4); Pain during activities: G1 > G2 (T4, *p* = 0.039; T5, *p* = 0.001); Walking distance: G1 > G2 (T5, *p* = 0.012); HSS total score: G1 > G2 (T5, *p* = 0.023); G1 = G2 (T6); G2 > G1 (T7).
Kawasaki T, 2009 [53]	Exercise program VAS + JKOM +	Hyaluronic acid VAS + JKOM +	—	—	VAS: G1 = G2 (T6, *p* = 0.171); JKOM: G1 = G2 (T6, *p* = 0.721); OMERACT-OARSI: G1 = G2 (T6, *p* = 0.767); Swelling: G1 > G2 (early OA, *p* = 0.019); G1 = G2 (advanced OA, *p* = 0.571); ROM: G1 = G2 (early OA, *p* = 0.879); G1 = G2 (advanced OA, *p* = 0.094). Biochemical markers: G1 = G2 (T6).

**Table 3:** Quality assessment using PEDro and RoB-2 scales. Quality assessment of six randomized controlled trials (RCTs) investigating graded motor imagery (GMI) and mirror therapy (MT) in patients with complex regional pain syndrome (CRPS) using the PEDro and RoB-2 Scales.

Study	PEDro Score (Total)	RoB-2: Bias in Randomization Process	RoB-2: Bias Due to Deviations from Intended Interventions	RoB-2: Bias Due to Missing Outcome Data	RoB-2: Bias in Outcome Measurement	RoB-2: Bias in Selection of the Reported Result	Overall RoB-2 Risk of Bias
Rezasoltani Z, 2020 [49]	08-ott	Low Risk	Low Risk	Low Risk	Low Risk	Low Risk	Low Risk
Saccomanno MF, 2016 [50]	07-ott	Low Risk	Some Concerns	Low Risk	Low Risk	Some Concerns	Some Concerns
Bao X, 2018 [51]	08-ott	Low Risk	Low Risk	Low Risk	Some Concerns	Some Concerns	Some Concerns
Karatosun V, 2009 [52]	06-ott	Some Concerns	Some Concerns	Low Risk	Low Risk	Low Risk	Some Concerns
Kawasaki T, 2009 [53]	06-ott	Low Risk	Some Concerns	Low Risk	Some Concerns	Some Concerns	Some Concerns

Legend: PEDro Score: Physiotherapy Evidence Database Score, RoB-2: Risk of Bias 2 Tool.

## Data Availability

Data are contained within the article.
